# ComE, an Essential Response Regulator, Negatively Regulates the Expression of the Capsular Polysaccharide Locus and Attenuates the Bacterial Virulence in *Streptococcus pneumoniae*

**DOI:** 10.3389/fmicb.2017.00277

**Published:** 2017-03-07

**Authors:** Yuqiang Zheng, Xuemei Zhang, Xiaofang Wang, Libin Wang, Jinghui Zhang, Yibing Yin

**Affiliations:** ^1^Department of Medicine Laboratory, Childrens Hospital of Chongqing Medical UniversityChongqing, China; ^2^Key Laboratory of Diagnostic Medicine Designated by the Ministry of Education, Chongqing Medical UniversityChongqing, China

**Keywords:** ComE, transcription regulation, capsule, virulence, *Streptococcus pneumoniae*

## Abstract

The capsular polysaccharide (CPS) of *Streptococcus pneumoniae* is the main virulence factors required for effective colonization and invasive disease. The capacity to regulate CPS production at the transcriptional level is critical for the survival of *S*. *pneumonia*e in different host niches, but little is known about the transcription regulators of *cps* locus. In the present study, we isolated and identified the response regulator ComE, the master competence switch in transformation of *S. pneumoniae*, as a transcriptional regulator of *cps* locus by DNA affinity chromatography-pulldown, MALDI-TOF mass spectrometry (MS) and electrophoretic mobility shift assay (EMSA). Our results showed that phosphorylated mimetic of ComE (ComE^D58E^) bound specifically to the *cps* locus prompter *in vitro*, and phosphorylated ComE negatively impacted both *cps* locus transcription and CPS production attenuating the pneumococcal virulence *in vivo*. Compared with D39-WT strain, D39ΔcomE mutant exhibited much thicker capsule, attenuated nasopharyngeal colonization and enhanced virulence in both pneumonia and bacteremia models of Balb/c mice. Furthermore, it was demonstrated that CSP-ComD/E competence system involved in regulating negatively the CPS production during the progress of transformation in D39. Our CSP1 induction experiment results showed that the expression of ComE in D39-WT strain increased powerfully by 120% after 10 min of CSP1 induction, but the CPS production in D39-WT strain decreased sharply by 67.1% after 15 min of CSP1 induction. However, the CPS production in D39ΔcomE mutant was almost constant during the whole stage of induction. Additionally, we found that extracellular glucose concentration could affect both the expression of ComE and CPS production of D39 *in vitro*. Taken together, for the first time, we report that ComE, as a transcriptional regulator of *cps* locus, plays an important role in transcriptional regulation of *cps* locus and capsular production level.

## Introduction

*Streptococcus pneumoniae* (pneumococcus), a Gram-positive opportunistic pathogen residing on the human upper respiratory tract, remains a leading cause of morbidity and mortality worldwide in young children and in immunocompromised elderly (Ostroff, 1999). The capsular polysaccharide (CPS) of *S*. *pneumoniae* is the key virulence factors required for effective colonization of the nasopharyngeal tract of the host and invasive infections in the blood and lungs (Kadioglu et al., 2008). Capsule is essential to pneumococcal virulence and their capacity to resist phagocytosis. Actually all of clinical isolates from sterile niches are encapsulated, while mutated non-encapsulated derivatives of these strains are mainly avirulent (Guildolin et al., 1995; Lanie et al., 2007; Hyams et al., 2010). *S*. *pneumoniae* requires a coordinated regulation in the expression of capsule to survive in different host niches. To colonize on pharynx nasalis, *S*. *pneumoniae* decreases the CPS production to facilitate exposure of important pneumococcal surface structures, such as adhesins, which aid in colonization. Once the pathogen escapes the nasopharynx and invades into lung and blood, maximal expression of capsule is essential to mask potential surface antigens, reduce complement deposition and protect the bacterium against opsonophagocytosis (Ogunniyi et al., 2002; Abeyta et al., 2003; Hammerschmidt et al., 2005). However, to date, very little is known about how to respond to specific environmental signals to alter the CPS expression in *S*. *pneumoniae*.

So far, at least 94 antigenically distinct serotypes of *S*. *pneumoniae* CPS are identified (Bentley et al., 2006; Park et al., 2007). All capsule types except for 3 and 37 serotypes are synthesized by the Wzy-dependent pathway, in which the CPS synthesis loci as a gene cluster are located at the same region of the chromosome between the *dexB* and *aliA* genes (Aanensen et al., 2007). The *cps* locus of the Wzy serotypes consists of the common genes (*cpsA*-*D*) and downstream type-specific genes (Figure S1A). The type-specific genes are responsible for CPS synthesis and export. The common genes (*cps*A-D) are highly homologous among serotypes of Wzy-dependent pathway, which seem to play a role in modulation of CPS synthesis. The proteins CpsA-D encoded by common genes have been shown to affect the level of CPS expression (Morona et al., 2000; Cieslewicz et al., 2001; Geno et al., 2014).

The ~87 bp region upstream of the initiation-codon of the *cpsA* gene, which contains the predicted −10 and −35 promoter motifs, has been turned out to be the only conserved sequence in all Wzy-dependent pathways in *S*. *pneumoniae* and contains a typical functional core promoter sequence (5′-TAGACA-17nucleotides-TATAAT-3′, *cps*p) (Munoz et al., 1997; Moscoso and Garcia, 2009). The core promoter is necessary for the full transcription of the capsule gene operon and for colonization and invasive diseases (Shainheit et al., 2014; Wen et al., 2015). Previous studies have demonstrated that mutation in the -10 box of the *cps* promoter from the consensus TATAAT to TACAAT or TATAAC results in marked reduced promoter strength and transcription of the *cps* genes associated with CPS formation (Lanie et al., 2007). In addition, several proteins involved in the regulation of sugar metabolism pathways, such as RegM, Pgm, and GalU, have been shown to influence transcription of the *cps* locus and capsular expression (Mollerach et al., 1998; Hardy et al., 2001; Giammarinaro and Paton, 2002). However, hitherto, the transcription factors of *cps* promoter remain uncharacterized, and the transcription regulation of CPS production is still poorly understood.

In this study, a 218 bp 5′ -biotin labeled DNA probe (upstream of the *cps2A*, contains 14 bp nucleotides downstream of *cps2A* initiation codon, Figure S1B) was utilized to isolate and identify the *cps* transcription factor by DNA affinity chromatography-pulldown, MALDI-TOF mass spectrometry (MS) and electrophoretic mobility shift assay (EMSA). In this way, seven proteins were initially chosen to be further studied as candidate transcriptional regulators of *cps* locus (Figure S2). We have performed in-depth molecular analyses of the response regulator ComE in this context. ComE regulates negatively the transcription of the *cps* locus gene and decreases the CPS production, which leads to attenuated virulence. We also found that CSP-ComD/E competence system involved in regulating negatively the CPS production in the development of competence D39, and extracellular glucose concentrations could impact the expression of ComE and regulate positively the CPS production. This is the first report that ComE, as a transcriptional regulator of the *cps* locus, plays an important role in regulating CPS production except for the master competence switch in natural genetic transformation of *S. pneumoniae*.

## Materials and methods

### Bacterial strains, plasmids, oligonucleotides, and growth conditions

All of the strains, plasmids and primers used in the present study are listed in Tables 1, 2. All of *S*. *pneumoniae* strains were grown at 37°C under 5% CO_2_ in C+Y (semisynthetic casein hydrolysate medium supplemented with 5% yeast extract) medium or on blood agar plates supplemented with 5% defibrinated sheep blood. *E. coli* was grown on LB agar plates or in LB broth with shaking at 37°C. All growth mediums were supplemented with appropriate antibiotics as described in Table 1. Procedures for transformation and competence development of *S*. *pneumoniae* were reported previously (Weng et al., 2013).

**Table 1:** **Bacterial strains and plasmids used in this study**.

**Strain/plasmid**	**Relevant properties**	**Antibiotic concentrations**	**Source or references**
***STREPTOCOCCUS PNEUMONIAE***			
D39			NCTC
*S. pneumoniae* CPM8	Erm^R^	0.25 μg/ml	Lee and Morrison, 1999
D39ΔcomE	Erm^R^	0.25 μg/ml	This study
D39::comE^*D*58*E*^	D39-*P_*czcD*_-gfp+*-comE^*D*58*E*^; Zn^2+^ -dependent production; Tet^*R*^	2.5 μg/ml	This study
D39ΔcomE::comE^*D*58*E*^	D39ΔcomE-*P_*czcD*_-gfp+*-comE^*D*58*E*^; n^2+^-dependent production; Tet^R^, Erm^R^	Tet^R^: 2.5 μg/ml Erm^R^: 0.25 μg/ml	This study
D39-p*EVP3*-cps-promoter	pEVP3, Chl^R^	2.5 μg/ml	This study
D39ΔcomE-p*EVP3*-cps-promoter	pEVP3-cps-promoter; D39ΔcomE; Erm^R^, Chl^R^	Erm^R^:0.25 μg/ml Chl^R^: 2.5 μg/ml	This study
D39ΔcomE-p*EVP3*-cps-promoter::comE^*D*58*E*^	D39ΔcomE-*P_*czcD*_-gfp+*-comE^*D*58*E*^; pEVP3-cps-promoter; D39ΔcomE; Erm^R^, Chl^R^; Tet^R^	Erm^R^:0.25 μg/ml Chl^R^: 2.5 μg/ml Tet^R^: 2.5 μg/ml	This study
D39-p*AE03*	pAE03; Erm^R^	0.25 μg/ml	This study
D39-p*AE03*-cps-promoter	pAE03-cps-promoter; Erm^R^	0.25 μg/ml	This study
D39ΔcomE-p*AE03*-cps-promoter	pAE03-cps-promoter; D39ΔcomE; ${\text{Tet}}_{2}^{\text{R}}$; Erm^*R*^	${\text{Tet}}_{2}^{\text{R}}$:0.3 μg/ml Erm^R^:0.25 μg/ml	This study
***ESCHERICHIA COLI***			
*E. coli* BL21(DE3)	Expression strain		Takara
*E. coli* DH5a	Cloning strain		Takara
**PLASMIDS**			
pJWV25	Zn^2+^-dependent production; Tet^R^	Tet^R^:12.5 μg/ml(in *E. coli*)	Eberhardt et al., 2009
pEVP3	Chl^R^	20 μg/ml(in *E. coli*)	Pestova and Morrison, 1998
pAE03	Erm^R^	50 μg/ml(in *E. coli*)	Eberhardt et al., 2009
pET-28a(+)	Kana^R^, (Expression vector)	50 μg/ml(in *E. coli*)	Takara
pJWV25::comE^*D*58*E*^	pJWV25 derivative, carrying comE^*D*58*E*^ fusion; Tet^R^	Tet^R^: 2.5 μg/ml	This study
pEVP3-cps-promoter	pEVP3 derivative, carrying cps promoter fusion; Chl^R^	20 μg/ml(in *E. coli*)	This study
pAE03-cps-promoter	pAE03 derivative, carrying cps promoter fusion; Erm^R^	50 μg/ml(in *E. coli*)	This study

*Erm^R^, Erythromycin^R^; Tet^R^, Tetracyclines^R^; Chl^R^, Chloromycetin^R^; Kana^R^, Kanamycin^R^*.

**Table 2:** **Primers used in this study**.

**Primer**	**Sequence (5′-3′)**	**Size (bp)**	**Description**
**PROBE**			
P*_*cps*_* F	Bio-TACACATCTGCTTCTAAAATATTGT	218	C2 labeled by biotin; 313542–313566 nt
P*_*cps*_* R	TTAAAACGTCTACTCATGATTAACA		313735–313760 nt
P*_*cps*_* F2	TACACATCTGCTTCTAAAATATTGT		Unlabeled probe C0; 313542–313566 nt
**ComE**			
comE-F	*CGGGATCC*ATGAAAGTTTTAGAA	750	*Bam*H I site
comE-R	*CCCTCGAG* TCACTTTTGAGATTTTTTCTC		*Xho* I site
**ComE**^**D58E**^			
comE^D58E^ –m2	CCATGAATATCGAT□C TCTAGGAAATAAAGC	193	comE mRNA _T174C_
comE^D58E^–m3	GCTTTATTTCCTAGA□G ATCGATATTCATGG	591	
**ΔcomE**			
comE-P1	AACATGCTCATCACAAAAGA	567	Upstream fragment
comE-P2	*ATCAAACAAATTTTGGGCCCGG*GATTGACAATTAGCAAGAA		
comE-P3	*ATTCTATGAGTCGCTGCCGACT*TTAAAACTTTCATTCAAATTC	642	Downstream fragment
comE-P4	ACACAGATGAAATTGTTGGT		
**OVER-EXPRESSION ComE**			
comE^D58E^-hb-F	*CTAGCTAGC*ATGAAAGTTTTAATTTTAGAA	775	*Nhe* I site
comE^D58E^-hb-R	*ATAAGAATGCGGCCGC*TCACTTTTGAGATTTTTTCTC		*Not* I site
p*EVP3*-cps-promoter			
p*EVP3*-cps-promoter-F	*GAAGATCT*AAGAAATCCTCTGATATCTTCTTCC	775	gI II site
p*EVP3*-cps-promoter-R	*TCCCCCGGG*CATGATTAACACCTATACATTGAAC		Sma I site
**pAE03-cps-PROMOTER**			
pAE03-cps-promoter-F	*CCGGAATTC*AAGAAATCCTCTGATATCTTCTTCC	783	EcoR I site
pAE03-cps-promoter-R	*ATAAGAATGCGGCCGC*CATGATTAACACCTATACATTGAAC		*Not* I site
**cps2A**			
cps2A-F	CGTCAACCGAAGCACTG		Real-time PCR
cps2A-R	GATCCATCCGACCTGTCC		
**16s rRNA**			
16s rRNA-F	GTAGTCCACGCTGAAACGATGATG		Real-time PCR
16s rRNA-R	CTGTCCCGAAGGAAAACTCTATCT		
**Erm**			
Erm-F	CCGGGCCCAAAATTTGTTTGAT	780	Erm^R^ marker
Erm-R	AGTCGGCAGCTCATAGAAT		
**Tet**_**2**_			
Tet_2_-F	CCGGGCCCAAAATTTGTTTGAT		${\text{Tet}}_{2}^{\text{R}}$ marker
Tet_2_-R	TCCCAAAGTTGATCCCTTAACGA		
**GFP**			
gfp-F	AAAGGAGAAGAACTTTTCACTGGAG	165	
gfp-R	AGTAGTGACAAGTGTTGGCCATGGA		

*Restriction enzyme sites are underlined*.

### Construction of ComE^D58E^ strains

To exhibit the activity of ComE protein *in vitro*, a phosphorylated mimetic mutant, ComE^D58E^, was constructed by site-directed mutagenesis of *comE* (Horton et al., 1990; Martin et al., 2013). Briefly, Two couples of PCR reactions (with primer pairs of *comE*-F and *comE*^D58E^-m2, *comE*^D58E^-m3, and *comE*-R) were used to amplified 193-bp and 591-bp partial complement DNA fragments from chromosomal DNA in D39-WT, which contained the mutagenesis site of *comE* DNA_T174C_. The two DNA fragments were fused by PCR (with primer pairs of *comE*-F and comE-R) to generate complete *comE*^D58E^ gene fragment, then was digested with *Bam*H I and *Xho* I, and ligated to *Bam*H I-*Xho* I-digested *p*ET-28a (+) plasmid DNA. Then *p*ET-28a-comE^D58E^ plasmid was transformed into *E. coli* B21 to generate the recombination *comE*^D58E^ expression strain. The *comE*^D58E^ DNA fragment was amplified by PCR with the primer pairs of comE^D58E^-hb-F and comE^D58E^-hb-R, and the PCR product was digested with *Nhe* I and *Not* I, subsequently, was ligated to Spe I- *Not* I-digested *p*JWV25 plasmid DNA. Then *p*JWV25::comE^D58E^ plasmid was transformed into D39 strain to generate the ComE over-expression strain D39::comE^D58E^ (Eberhardt et al., 2009).

### Construction of series of D39 mutants

The D39 (serotype 2; NCTC) was used as wild type (WT) strain. To construct the D39ΔcomE and D39ΔcomE::comE^D58E^ mutants, which derived from D39 and D39::comE^D58E^ strain, respectively, the 567-bp upstream and 642-bp downstream DNA flank of comE were amplified by PCR (with primer pairs of comE-P1 and comE-P2, comE-P3 and comE-P4). 780-bp Erm gene fragment was acquired by PCR from *S. pneumoniae* CPM8 strain (with primer pairs of Erm-F and Erm-R). To acquire the transformation donor DNA, a third PCR was carried out to fuse the upstream-Erm-downstream fragment with primer pairs of comE-P1 and comE-P4. The *comE* gene was completely replaced with the erythromycin gene by homologous recombination according to an established protocol (Wu et al., 2010).

### Transcriptional reporter constructs, β-galactosidase and GFP assay

To detect the effect of ComE on the transcription of *cps* promoter, β-galactosidase and GFP reporter constructs of *cps* promoter region were prepared in the pEVP3 and pAE03 plasmid vectors, respectively. A *cps* promoter fragment containing 780-bp upstream of *cps2A* was cloned into pEVP3 (at the BgI II and Sma I restriction sites) and pAE03 (at the EcoR I and *Not* I restriction sites), respectively. The pEVP3-cps-promoter and pAE03-cps-promoter plasmids were then transformed into D39-WT strains to generate the D39-*pEVP3*-cps-promoter and D39-*pAE03*-cps-promoter mutants. D39ΔcomE-p*EVP3*-cps-promoter and D39ΔcomE-p*AE03*-cps-promoter mutants were constructed by homologous recombination according to an established protocol (Wu et al., 2010). β-galactosidase and GFP assay were performed as described previously (Pérez et al., 2008; Eberhardt et al., 2009). All assays were triplicate, and the results of representative experiments are presented as means of three replicates ± standard deviations.

### DNA affinity chromatography-pulldown

To isolate and identify the transcription factors of *cps* promoter in *S*. *pneumoniae*, a 5′ biotin modification 218-bp DNA probe (C2 probe;313542-313760 nt; 400 ng/μl) was PCR amplified from the chromosomal DNA of D39-WT strain using the primer pairs of P_*cps*_ –F and P_*cps*_–R (Moscoso and Garcia, 2009; Jutras et al., 2010). Procedures for DNA affinity chromatography were reported previously (Jutras et al., 2012). Briefly, fresh cells lysate preparation (harvested from 100 ml of a D39 culture medium induced with 10 μg/ml CSP1 at an OD_600_ of 0.3; 250 μg/ml) was carried out by sonication with freeze thaw. The cells lysate was then incubated with C2-probe-streptavidin-coated magnetic beads (DynabeadsM-280 Streptavidin; Invitrogen, Darmstadt, Germany) and purified with different NaCl concentration elution buffer, using the blank streptavidin-coated magnetic beads without C2 probe-coated as the control group. The elution protein under different NaCl elution buffer was collected respectively, analyzed via 15% SDS-PAGE, and stained by SYPRO-Ruby. Compared with control group, the enriched proteins were detected, excised and identified by MAIDI-TOF mass spectrometry (BGI, Beijing Genomics Institute, China).

### Cloning, expression and purification of soluble ComE and ComE^D58E^ proteins

To construct expression plasmids, *comE* and *comE*^D58E^ gene were PCR amplified from chromosomal DNA of D39-WT and constructed *comE*^D58E^ mutant fragment with the primer pairs of comE-F and comE-R, respectively. The PCR product was purified and digested with *Bam*H I and *Xho* I, and ligated to *Bam*H I-*Xho* I-digested *p*ET-28a(+) plasmid DNA. The *E. coli* B21 (DE3) strain was then successively transformed with a *p*ET-28a-comE or *p*ET-28a-comE^D58E^. Expression and purification of ComE and ComE^D58E^ proteins were then carried out as reported previously (Martin et al., 2013). Briefly, *E. coli* was grown at 37°C in 1000 ml of LB medium, supplemented with 50 μg/ml Kanamycin, with 180 rmp shaking for 6 h, and ComE or ComE^D58E^ expression was induced with 0.5 mM IPTG (Sigma) at an OD_600_ of 0.8 with 120 rmp shaking at 20°C for 18–20 h. Cells were harvested by centrifugation at 10,000 × g for 30 min at 4°C, resuspended in 50 ml of binding buffer, and lysised completely by sonication. Soluble His-tagged ComE or ComE^D58E^ proteins retained on Ni-NTA column were then eluted with increasing concentration imidazole buffer. The enriched proteins were analyzed for purity by SDS-PAGE and concentrated by ultrafiltration, and then small packing stored at −20°C.

### Electrophoretic mobility shift assay (EMSA)

The upstream promoter regions of *cps*2A in D39 (218 bp) was PCR amplified to produce the 5′ biotin modification labeled probe C2 and unlabeled probe C0 with primer pairs of P_*cps*_-F/P_*cps*_-R and P_*cps*_–F2/P_*cps*_–R, respectively. The PCR product was purified with the high pure PCR product purification kit (Cat. no.11 732 676 001, Roche). EMSA was carried out according to the protocol of LightShift® Chemiluminescent EMSA Kit (number 20148, Thermo). Briefly, DNA-binding assays were incubated at 37°C for 30 min in 20 μl reaction buffer containing 1 × binding buffer, 1 μg Poly (dI-dC), 2.5% Glycerol, 0.05% NP-40, 5 mM Mn^2+^, 5 mM Zn^2+^, 2–4 μg interest proteins, 1 ng labeled probe C2 or 100 ng unlabeled probe C0. Following incubation, binding reaction mixtures were analyzed by electrophoresis in 6% native TBE polyacrylamide gels at constant 100 V for 60 min. Then the gels were transferred to nylon membrane at 380 mA for 40 min, and crosslinked for 10 min by a hand-held UV lamp equipped with 254 nm bulbs, and subsequently biotin-labeled DNA binding were detected by chemiluminescence according to supplier's instructions.

### Morphological observation of capsule

To compare the difference of capsule between D39-WT and D39ΔcomE mutant, Neufeld test, immunofluorescence microscopy and transmission electron microscopy (TEM) were carried out according to the method described earlier (Klimenko and Baturo, 1980; Lu et al., 2006). Briefly, Neufeld test: the culture of D39-WT and D39ΔcomE mutant were spread on a slide respectively. The rabbit anti-serotype-2 serum (States Serum Institut, Denmark, at dilutions of 1:1,000) was added onto the slide, placed in a wet box at 37°C for 25 min. The slide was taken out and dried naturally, and stained with 1% methylene and 1% India ink stain. Under the oil immersion, the count of *S*. *pneumoniae* was taken and the microscopic surveying instrument was used to determine the capsule thickness of 100 randomly chosen capsule swelling cells.

Immunofluorescence microscopy: CPS of D39-WT and D39ΔcomE mutant were detected by immunofluorescence microscopy as described earlier (Lu et al., 2006), except using different primary antiserum and fluorescent labeling secondary antibody. In this study, rabbit anti-serotype-2 serum and Dylight594-conjugated goat anti-rabbit IgG (Bio-Rad, at dilutions of 1:3,000) were used.

TEM: Cells were grown to an OD_600_ of 0.3 in 5 ml of C+Y at 37°C under 5% CO_2_, and harvested by centrifugation at 10,000 × g for 15 min at 4°C. The cell pellets were fixed in 2% glutaraldehyde in sodium cacodylate buffer for 24 h, and processed by the Electron Microscopy Research Service of Chongqing Medical University. Samples were sectioned and examined using a Hitachi H-7500 transmission electron microscope. Capsule thickness was determined by measuring 20 randomly chosen cells by Image J software.

### RT-PCR assays

Cells were grown to an OD_600_ of 0.3 in 5 ml C+Y medium at 37°C under 5% CO_2_, and harvested by centrifugation at 4°C for 15 min at 10,000 × g. The Trizol reagent (Invitrogen, Beijing, China) was used to isolated total RNA from D39, and the cDNAs of D39 were prepared using the total RNA preparation with an iScript™ cDNA synthesis kit (Bio-Rad, Beijing, China) according to the supplier's instructions. The mRNA level of the *cps* locus was quantified by quantitative real time PCRs using cDNA as the template and the primer pairs of *cps*2A-F/*cps*2A-R and 16S rRNA-F/16S rRNA-R (as an internal control for constitutively expressed gene) as described (Shainheit et al., 2014). The relative expression level of *cps*2A was calculated using the average mean cycle threshold value for the *cps*2A of each sample and normalized with that of 16S rRNA. The results of representative experiments were presented as means of three replicates ± standard deviations.

### CPS analyses

Relative CPS levels of D39-WT, D39ΔcomE, D39ΔcomE**::**comE^D58E^, and D39::comE^D58E^ mutants were determined using Western blot analysis and enzyme-linked immunosorbent assay (ELISA) as described previously (Xayarath and Yother, 2007; Geno et al., 2014). Briefly, D39-WT, D39ΔcomE and D39ΔcomE::comE^D58E^ mutants were grown to an OD_600_ of 0.3 in 5 ml C+Y medium supplemented with 0.10 mM Zn^2+^ at 37°C under 5% CO_2_. D39::comE^D58E^ mutants were grown in 5 ml C+Y medium supplemented with increasing Zn^2+^ concentration from 0 to 0.15 mM, respectively. The cells were harvested by centrifugation at 4°C for 5 min at 10,000 × g. The lysis of cell pellets was performed using 10% sodium deoxycholate, and samples concentration were standardized to 0.8 mg/ml by total protein quantification (Bio Rad Protein Assay, Bradford method).

For Western blot analysis, CPS was detected with a rabbit anti-serotype-2 serum (at dilutions of 1:2,000) as primary antibody and peroxidase-conjugated goat anti-rabbit IgG as secondary antibody (Bio-Rad, at dilutions of 1:5,000), respectively. The member of the CPS-antibody was visualized using Western ECL substrate (Bio-Rad) according to the supplier's instructions. Quantification was done by spot densitometry using the Image J software (Image J 1.47v, National Institutes of Health, USA). The results of representative experiments were presented as means of three replicates ± standard deviations.

For ELISA analysis, as described previously (Xayarath and Yother, 2007). Briefly, samples were prepared and standardized as described above. Dilution samples of 1:800 were used to coat 96-well microtiter plates, which were subsequently reacted with the rabbit anti-serotype-2 serum, at dilutions of 1:3,000, which had been absorbed with the CPS-negative strain. The bound primary antibodies were detected following incubation with goat anti-rabbit IgG-HRP (Bio-Rad) at dilutions of 1:5,000, and the absorbance at 450 nm was recorded. The ratio of absorbance value at 450 nm to total protein concentration of each sample was calculated. The results of representative experiments were presented as means of three replicates ± standard deviations.

### Induction expression of ComE by CSP1

To further verify the effect of comE on the transcription of *cps* locus promoter, the induction expression of ComE was carried out using exogenous CSP1 as inducer (CSP1, competence-stimulating peptide, was synthesized by the Shanghai Sheng-gong Company China; CSP1:H-Glu-Met-Arg-Leu-Ser-Lys-Phe-Phe-Arg-Asp-Phe-IIe-Leu-Gln-Arg-Lys-Lys-OH;1 mg/ml).D39-p*AE03*-cps-promoter and D39ΔcomE-p*AE03*-cps-promoter mutants were grown in 30 ml C+Y medium supplemented with 1 mmol CaCl_2_ and 2% g/L BSA at 37°C under 5% CO_2_, respectively. When the culture reached an OD_600_ of about 0.1, cells were induced to the competence by CSP1 (500 ng/ml). 5 ml sample was taken periodically out from the culture after induction: 0, 5, 10, 15, 20 and 25 min. Samples were taken out and chilled rapidly on dry ice, without freezing, and then kept at 4°C until harvested by centrifugation at 10,000 × g for 5 min at 4°C. ComE and GFP were detected by Western blot analysis as described above. Glyceraldehyde-3-phosphate dehydrogenase (GAPDH) was detected as an internal control for constitutively expressed gene. D39-p*AE03* strains were used as control strains which were transformed with blank p*AE03* plasmid.

D39 and D39ΔcomE mutant were induced by CSP1 as described above. ComE and CPS were detected by ELISA. Samples for ComE were standardized to 0.8 mg/ml by total protein quantification, and then were used to coat 96-well microtiter plates at dilutions of 1:200, which were subsequently reacted with the mouse anti-ComE serum at dilution of 1:800. The bound conjugate were detected following incubation with goat anti-mouse IgG-HRP (Bio-Rad) at dilutions of 1:5,000, and the absorbance value at 450 nm was recorded. The results of representative experiments were presented as means of three replicates ± standard deviations.

### Effect of glucose concentration on the expression of ComE and CPS production

D39-WT and D39ΔcomE strains were grown at 37°C under 5% CO_2_, to an OD_600_ of 0.3 in 5 ml C+Y medium supplemented with following different concentration glucose: 2.0, 4.0, 8.0 mM (the glucose concentration in C+Y medium) and 16.0 mM, respectively. The samples were harvested and prepared for analysis as described above. The ComE and CPS were detected by Western blot and ELISA, respectively. The results of representative experiments were presented as means of three replicates ± standard deviations.

### Animal studies

All of the animals used in this study were purchased from the Department of Experimental Animal, Chongqing Medical University [certificate no. SYXK(yu) 2007-0001]. All animal procedures were approved by the Ethics Committee of Chongqing Medical University (reference no.2011-032). The research described here was carried out in accordance with the Declaration of Helsinki and with the recommendations in the Guide for the Care and Use of Laboratory Animals of the National Institutes of Health.

For nasopharyngeal colonization model: D39-WT and D39ΔcomE mutant were grown in C+Y medium to an OD_600_ of 0.5 at 37°C under 5% CO_2_. The cells were adjusted to 3.0 × 10^6^ CFU/30 μl concentration. Groups of two 6-week-old female Balb/c mice (*n* = 18) were intranasally challenged with 30 μl D39-WT or D39ΔcomE mutant. The animals were killed after inoculation: 6, 12, 24, 36, 48, and 72 h. Colonized bacteria were harvested from nasal lavage fluid and enumerated by plating serial dilutions. The nasopharyngeal lavage was collected as described previously (Wu et al., 1997). The results of representative experiments were presented as means of three replicates ± standard deviations.

For a pneumonia model of infection: D39-WT, D39ΔcomE and D39::comE^D58E^ mutants were grown as described above. The cells were resuspended in PBS and adjusted to 7.5 × 10^7^ CFU/30 μl concentration. Groups of three 6-week-old female Balb/c mice (*n* = 12) were intranasally challenged with 30 μl D39-WT, D39ΔcomE or D39::comE^D58E^ mutant as described earlier (Wu et al., 1997). Mice were subsequently monitored for 8 days, and survival was recorded every day.

For a bacteremia model of infection: D39-WT, D39ΔcomE and D39::comE^D58E^ mutants were grown as described above. The cells were resuspended in PBS and adjusted to 7.5 × 10^2^ CFU/50 μl concentration. 50 μl of D39-WT, D39ΔcomE or D39::comE^D58E^ mutants were administered to 6-week-old female Balb/c mice (*n* = 12) by intraperitoneal injection, respectively. Mice were subsequently monitored for 4 days, and survival was recorded every 12 h.

### Statistical analysis

Differences between groups were statistically analyzed with a two-tailed *t*-test or the non-parametric Mann-Whitney U-test or Gehan-Breslow-Wilcoxon test with GraphPadPrism 5 (GraphPad Software, San Diego, CA). *P* < 0.05 was considered to be statistically significant.

## Result

### Response regulator ComE was isolated and identified using CPS promoter DNA as bait

**Figure 1:**
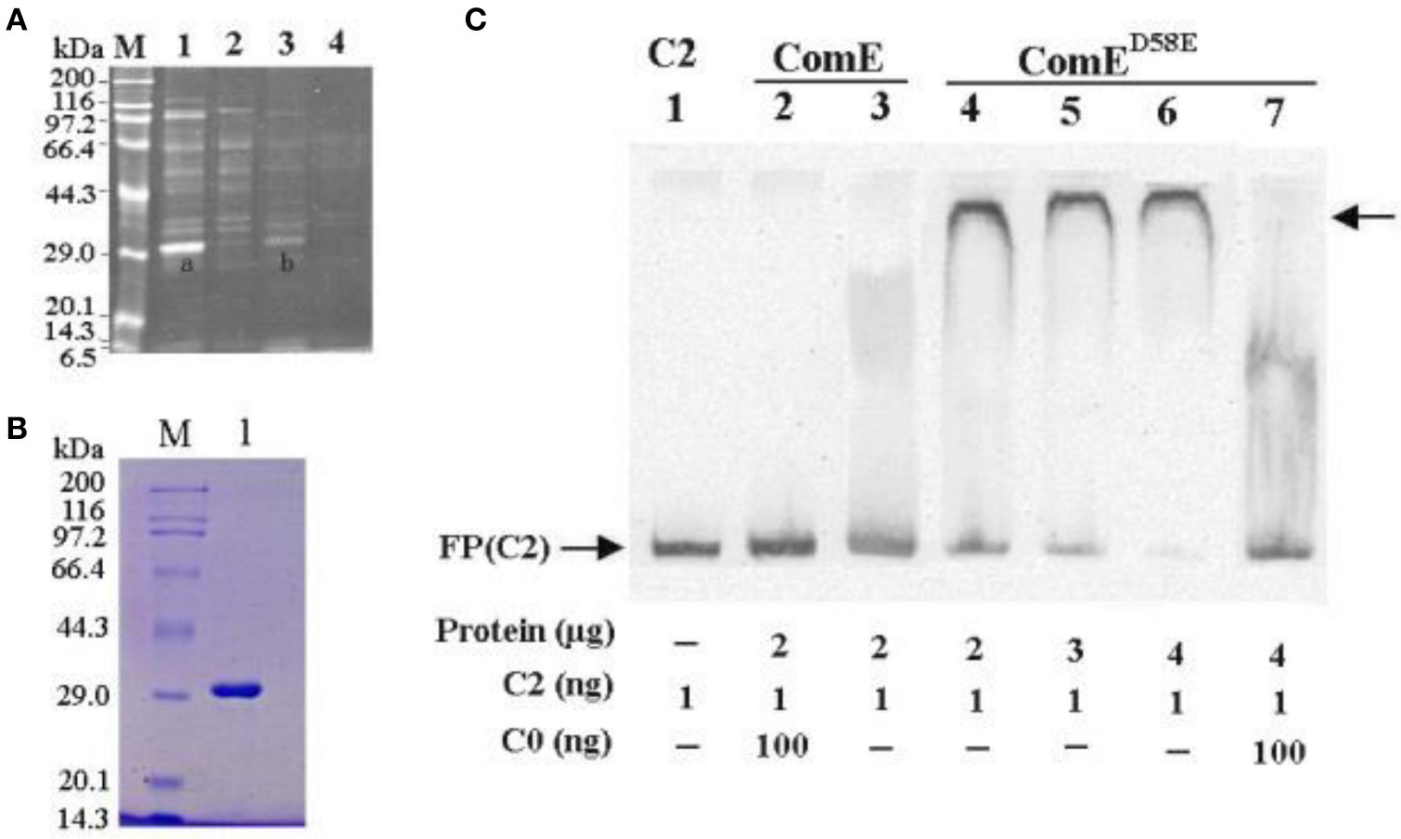
**Isolating and identifying response regulator ComE using ***cps*** promoter DNA as bait. (A)** SDS-PAGE analysis of *S. pneumoniae* cytoplasmic proteins fished out by affinity chromatography using *cps* promoter DNA probe (C2) as bait. The enriched proteins were excised and identified by MAIDI-TOF mass spectrometry, proteins bands shown as **(A,B)**. Numbers on the left indicate positions of molecular mass standards. **(B)** Soluble ComE and ComE^D58E^ proteins were expressed and purified. The molecular weight of ComE and ComE^D58E^ is about 29.9kDa, shown in Lane 1. **(C)** EMSA demonstrated that ComE^D58E^ binded specifically to the C2 probe, but ComE not to C2 probe. C0 indicated unlabeled probe which binded competitively to the ComE or ComE^D58E^ with the biotin-labeled probe C2. The experiments were repeated three times with similar results. A representative gel image was shown.

Earlier work has demonstrated that the genes in *cps* locus are co-transcribed as an operon from a common promoter upstream of *cps*A (Guildolin et al., 1995; Shainheit et al., 2014; Wen et al., 2015). A bioinformatic analysis showed that the ~250 bp region located immediately upstream of *cps*A was relatively conserved in the most *S. pneumonia*e isolates synthesizing their capsular through a Wzy-polymerase-dependent mechanism (Moscoso and Garcia, 2009). In this conserved region, there are many potential and putative transcriptional regulators binding sites such as ComX1(Luo and Morrison, 2003), CopY (Reyes et al., 2006), MalR (Nieto et al., 1997), GlnR (Kloosterman et al., 2006), and RitR (Ulijasz et al., 2004). However, the putative transcriptional regulator of the *cps* gene has still not been experimentally demonstrated. In this study, we screened the transcriptional regulator of the *cps* gene cluster in the D39-WT strain by DNA affinity chromatography-pulldown, MALDI-TOF MS analysis and EMSA using 218 bp DNA probe (C2) that located immediately upstream of *cps*A. We obtained an enriched protein at about 29 kDa molecular mass in SDS-PAGE, which was subsequently identified as response regulator ComE with 78.4% coverage rate by MALDI-TOF MS analysis (Figures 1A,B).

However, the EMSA results showed that the ComE could not bind specifically the C2 probe because the purified recombination ComE was not phosphorylated by ComD *in vitro*. To exhibit the binding activity of ComE~P *in vitro*, a phosphorylated mimetic mutant, ComE^D58E^, was further constructed by site-directed mutagenesis of comE gene (Martin et al., 2013). The ComE^D58E^ exhibited significantly improved binding affinity for C2 probe by EMSA (Figure 1C). Overall, these results indicated that non-activated ComE could bind hardly to the *cps* promoter sequence, but phosphorylated mimetic ComE^*D*58*E*^ could exhibit significantly binding affinity for it. It implies that the phosphorylation of ComE may be very important to its binding to *cps* promoter.

### ComE impacts on the capsule production by down-regulation of the CPS locus transcription

**Figure 2:**
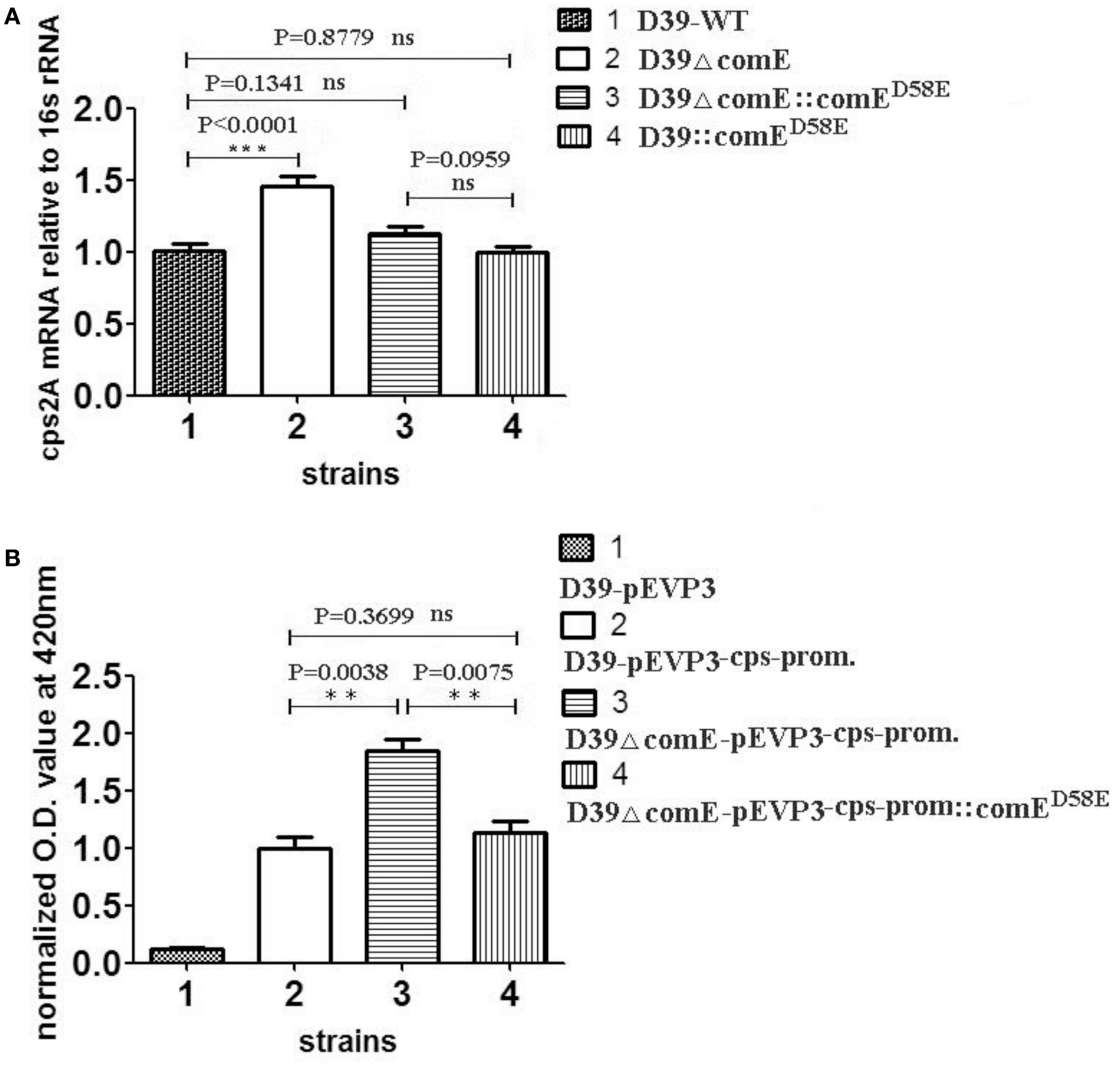
**ComE impacts on the ***cps*** transcription. (A)** Quantification of the *cps2A* mRNA by qRT-PCR in D39 and its mutants. The amount of the *cps2A* PCR product in each sample was quantified by its average mean cycle threshold value and normalized with that of the 16srRNA in the same reaction. **(B)** Impact of ComE on transcription activity of the *cps* promoter as assessed by a beta-galactosidase reporter. The results of representative experiments are presented as means of three replicates ± standard deviations. ^**^P < 0.01; ^***^P < 0.0001; ns, no significant difference.

Previous studies have demonstrated that the ComD/E pair is a classical two-component signal transduction system (TCS), which constitutes, together with competence-stimulating peptide (CSP), the master competence switch (Pestova et al., 1996; Gao and Stock, 2009; Martin et al., 2010; Weng et al., 2013). The *S. pneumoniae* ComD/E not only controls the development of genetic competence in the bacterium but also affects virulence in models of bacteremia and pneumonia by regulating a variety of virulence factors directly or indirectly (Ibrahim et al., 2004; Guiral et al., 2005; Kowalko and Sebert, 2008). The CPS is the critical virulent factor required for effective colonization for *S*. *pneumoniae* in the pharynx nasalis and invasive infections in the blood and lungs (Kadioglu et al., 2008). However, so far, it has not been reported that ComE can regulate the expression of CPS. Our results above showed that phosphorylated mimetic ComE^*D*58*E*^ can bind specifically to the *cps* promoter *in vitro*, which hinted that the ComE might affect the transcription of *cps* locus. To determine the effect of ComE on the *cps* transcription, we constructed D39ΔcomE, D39::comE^D58E^, D39ΔcomE::comE^D58E^, D39-p*EVP3*-cps-promoter, and D39ΔcomE-p*EVP3*-cps-promoter mutants in D39. The effect of each mutant on the *cps* transcription was assessed by qRT-PCR and β-galactosidase activity analysis, respectively. As compared with D39-WT strain, the level of transcription of *cps2A* in D39ΔcomE mutant increased approximately by 44.4% (^***^p < 0.0001), but the difference disappeared when the deleted *comE* gene was ectopic complemented in D39ΔcomE::comE^D58E^ (ns, *p* = 0.1341; Figure 2A).

The β-galactosidase activity analysis of D39-p*EVP3*-cps-promoter and D39ΔcomE-p*EVP3*-cps-promoter were carried out to assess the effect of ComE on the transcription of *cps* locus. The results showed that the β-galactosidase activity of D39ΔcomE-p*EVP3*-cps-promoter mutant increased significantly by 84.1% as compared with that of D39-p*EVP3*-cps-promoter (^**^p = 0.0038), and there was no significant differences between D39-p*EVP3*-cps-promoter and D39ΔcomE-p*EVP3*-cps-promoter::comE^D58E^ (ns, *p* = 0.3699, Figure 2B). These results indicated that the *comE* gene involved in the transcription of the *cps* locus in D39.

**Figure 3:**
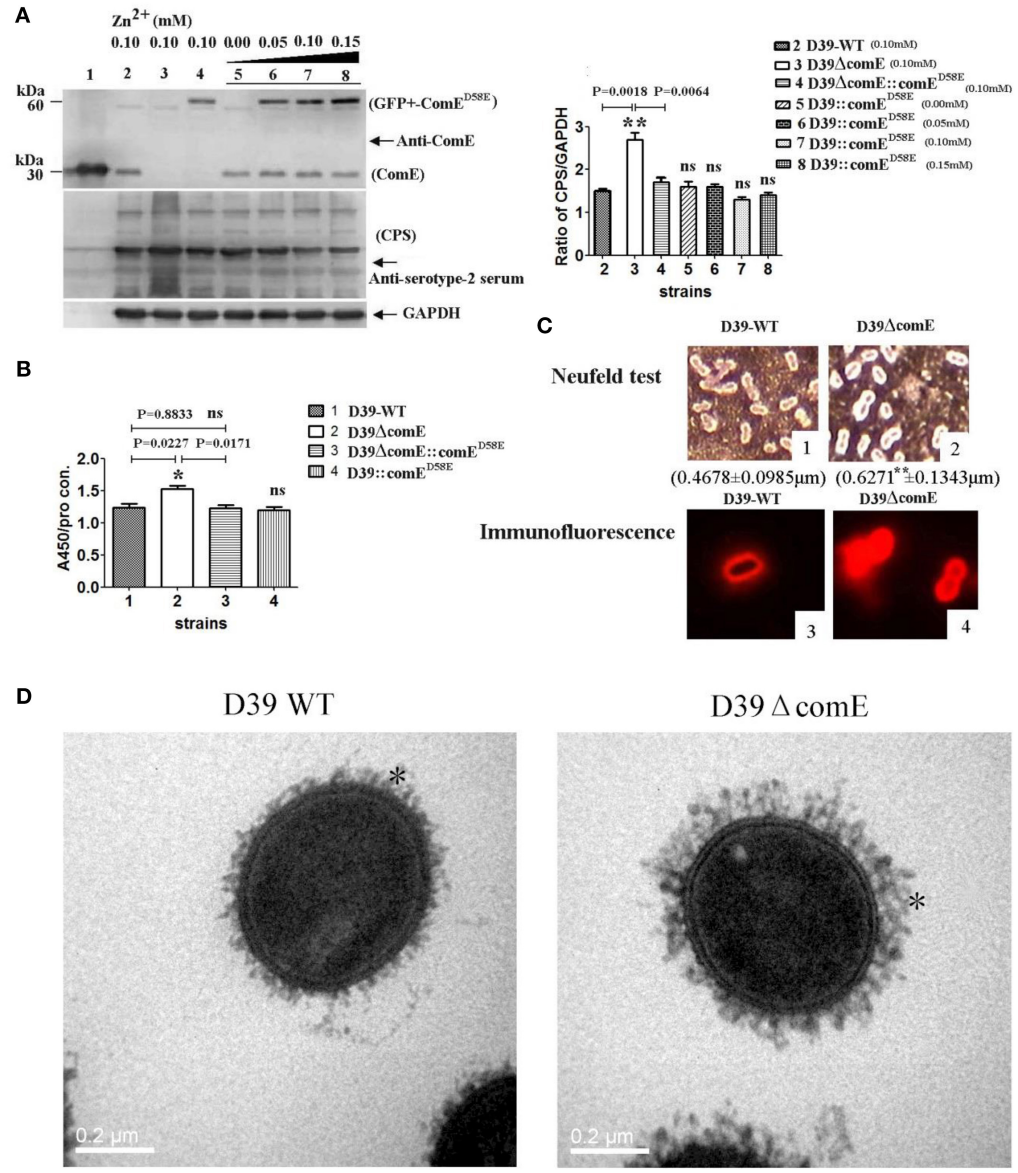
**ComE impacts on the CPS production. (A)** Detection of CPS production and ComE protein in D39 and its mutants by Western blot. ComE and GFP+-ComE^D58E^ were detected using anti-ComE antibodies. Fine control of GFP+-ComE^D58E^ expression using the Zn^2+^-inducible *P*_CZCD_ promoter by the addition of Zn^2+^. CPS was detected using anti-serotype-2 serum. GAPDH was detected as an internal control for constitutively expressed gene. Lanes: 1, soluble His-tagged ComE; 2.D39-WT; 3.D39ΔcomE; 4.D39ΔcomE::comE^D58E^; lanes 5–8, D39::comE^D58E^. Cells were grown in C+Y medium supplemented with the indicated concentrations of Zn^2+^. **(B)** Detection of CPS production in D39 and its mutants by ELISA. The ratio of absorbance value at 450 nm to total protein concentration in each sample was calculated. The results of representative experiments are presented as means of three replicates ± standard deviations, ^*^P < 0.05. **(C)** 1 and 2 showed the Neufeld test. The mean capsule thickness from 100 swelling cells was measured with Image J software, D39-WT (0.4678 ± 0.0985 μm) and D39ΔcomE mutant (0.6271 ± 0.1343 μm, ^**^P < 0.01); 3 and 4 showed the capsule stained using immunoflouorescence. The D39ΔcomE strains exhibit stronger immunoflouorescence than that of D39-WT strains. **(D)** Representative TEM images of D39-wt and D39ΔcomE mutant. Capsules are indicated with asterisks. The average capsule thickness of D39-WT and D39ΔcomE is 21.43 ± 0.4933 nm and 37.57 ± 0.5214 nm, respectively; ^**^P < 0.01). The bar (magnification is 80,000) indicates 200 nm.

We also assessed the role of the *comE* gene on the capsule production of D39 by Western blot and ELISA. The Western blot result showed that the CPS production of D39ΔcomE mutant increased significantly approximately by 80% (^**^p = 0.0018) and 58.8% (^**^p = 0.0064) as compared with that of D39-WT and D39Δ*comE*::*comE*^*D*58*E*^, respectively, and there was no significant differences between complemented D39ΔcomE::comE^D58E^ mutant and D39- WT strains (*p* = 0.1963, Figure 3A). The ELISA results demonstrated that the CPS production of D39Δ*comE* mutant increased significantly approximately by 23.0% (^*^p = 0.0227) and 24.3% (^*^p = 0.0171) as compared with that of D39-WT strains and D39ΔcomE::comE^D58E^ mutants, respectively. There was no significant difference between D39-WT strain and D39ΔcomE::comE^D58E^ mutant (ns, *p* = 0.8833; Figure 3B). These results indicated that the deletion of ComE can increase the CPS production of D39.

To further confirm our observations, we surveyed the capsule morphology difference between D39-WT strain and D39ΔcomE mutant by Neufeld test, immunofluorescence microscopy and transmission electron microscopy (TEM). The D39ΔcomE mutant exhibited stronger immunofluorescence and much thicker capsule than that of D39-WT strain (Figures 3C,D). The mean capsules thickness for D39ΔcomE mutant was 0.6271 ± 0.1343 μm (for Neufeld test) and 37.57 ± 0.5214 nm (for TEM), and for D39-WT strain 0.4678 ± 0.0985 μm (for Neufeld test) and 21.43 ± 0.4933 nm (for TEM), respectively. Statistical analysis revealed that the average capsular thickness of D39ΔcomE mutant was significantly increased compared with that of D39-WT strain (^***^p < 0.001).

Taken together, all these data has demonstrated that the ComE is a transcriptional regulator of *cps* locus and negatively regulates the CPS production.

### CSP-ComD/E competence system involved in regulating negatively the CPS production in the development of genetic competence of D39 strain

**Figure 4:**
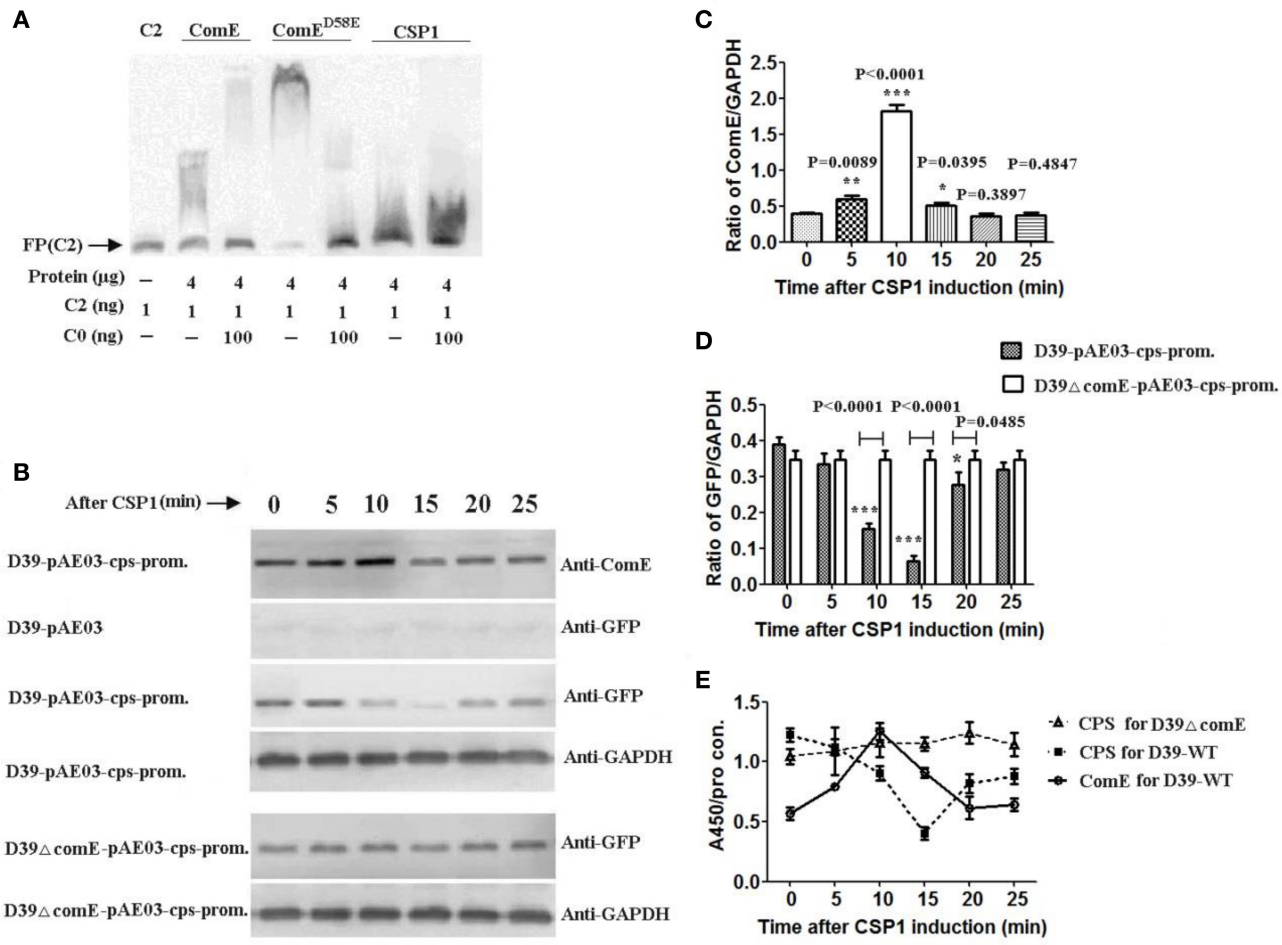
**CSP-ComD/E competence system involved in regulating negatively the CPS production in D39 strain (A)** EMSA of ComE, ComE^D58E^, and CSP1 binding to the C2 probe. The results show that CSP1 can't bind specifically to the C2 probe. **(B)** Impact of CSP1 on comE expression and transcription activity of the *cps* promoter as assessed by a GFP reporter for *cps* gene. **(C)** Quantitative chemiluminescence intensity levels of ComE from the Western blot results represented in **(A)**. The gray values ratio of ComE to GAPDH was calculated. The results of representative experiments are presented as means of three replicates ± standard deviations. ^*^P < 0.05; ^**^P < 0.01; ^***^P < 0.0001. **(D)** Quantitative chemiluminescence intensity levels of GFP from the Western blot results represented in **(A). (E)** Detection of CPS production and ComE protein induced by CSP1 during the whole stage of induction by ELISA. The ratio of absorbance value at 450 nm to total protein concentration in each sample was calculated. The results of representative experiments are presented as means of three replicates ± standard deviations.

It is well documented that competence stimulating peptide (CSP) induces the transcription of ComD/E and virulence genes in *S. pneumonia*e (Ween et al., 1999; Peterson et al., 2004; Zhu and Lau, 2011). According to the CSP-ComD/E competence system model, at a critical CSP concentration, CSP binds to its receptor ComD, which in turn activates its cognate response regulator called ComE, to induce the expression of an early group of competence genes and virulent factors required for infection. At the same time, ComE acts as a transcription factor activating its own comCDE promoter, which forms a positive feedback regulation of ComE. Our results above showed that ComE could regulate negatively the expression of CPS in D39. Therefore, we hypothesized that the CSP-ComD/E competence system involved in regulation of the CPS production during transformation of *S. pneumoniae*. In this study, we used CSP1 as exogenous inducer to detect the impacts of CSP-ComD/E competence system on the capsule production. We initially assessed the binding ability of ComE, ComE^D58E^ and CSP1 to the *cps* promoter by EMSA *in vitro*, and denied the specific binding of CSP1 to the *cps* promoter (Figure 4A).

To determine the role of CSP1 on the transcription of *cps* locus, we constructed the GFP reporter bacterium for *cps* gene expression, D39-*pAE*03-cps-promoter and D39ΔcomE-*pAE*03-cps-promoter mutant. ComE and reporter protein GFP were detected by Western blot. The results showed that the expression of ComE in D39-*pAE*03-cps-promoter started to increase after CSP1 induction 5 min, and reached peak value which increased 3.38-fold higher after CSP1 induction about 10 min (compared with that of 0 min, ^***^P < 0.0001), and then decreased promptly after CSP1 induction 15–20 min (Figures 4B,C). Corresponding to the expression of ComE, the expression of reporter protein GFP in D39-*pAE*03-cps-promoter decreased sharply by 83.3% after CSP1 induction about 10–15 min (compared with that of 0 min, ^***^P < 0.0001), and then restored after about 25 min. However, the expression of GFP in D39ΔcomE-*pAE*03-cps-promoter was almost unaffected by CSP1 in the development of competence from 0 to 25 min (Figures 4B,D).

We further assessed the role of the CSP1-ComD/E competence system on the capsule production of D39 by ELISA. The results showed that the expression of ComE in D39-WT increased powerfully by 120% after CSP1 induction about 10 min, and the CPS production in D39-WT decreased sharply by 67.1% after CSP1 induction about 15 min. However, the CPS production in D39ΔcomE mutant is almost constant during the whole stage of induction (Figure 4E).

Overall, these results demonstrated that CSP-ComD/E competence system could regulate negatively the CPS production in the development of competence in D39 strain.

### Glucose concentration impacts on the expression of ComE and the CPS production in D39

**Figure 5:**
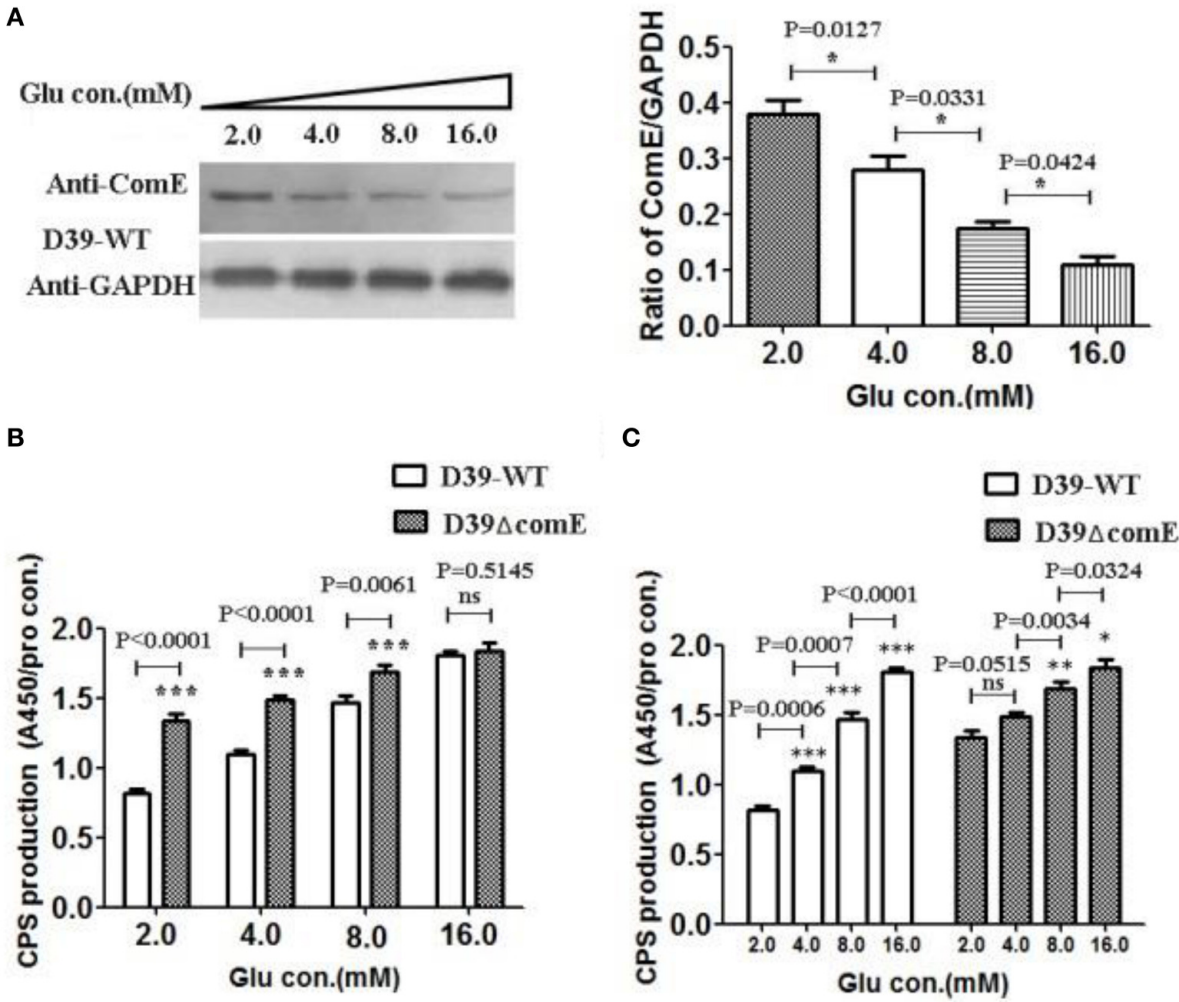
**Glucose concentration impacts on the expression of ComE and the CPS production in D39. (A)** Impact of the glucose concentration on the expression of ComE in D39-WT strain. D39-WT strains were grown to an OD_600_ of 0.3 in 5 ml of C+Y medium supplemented with different concentration glucose from 2.0 to 16.0 mM, and harvested by centrifugation, and detected by Western blot. GAPDH was detected as an internal control for constitutively expressed gene. The experiments were triplicate with similar results. The results of representative experiments are presented as means of three replicates ± standard deviations. ^*^P < 0.05. **(B,C)** Impact of the glucose concentration on the CPS production. CPS was detected by ELISA, and the ratio of absorbance value at 450 nm to total protein concentration in each sample was calculated. The results of representative experiments are presented as means of three replicates ± standard deviations. ^**^P < 0.01; ^***^P < 0.0001; ns, no significant difference.

It is clear that the capsular of *S. pneumonia*e isolated from the blood of infected mice is much thicker than that of the one grown *in vitro* (Ogunniyi et al., 2002). The glucose concentration is very low in healthy pharynx nasalis, where a reduced amount of CPS is required for optimal adhesion of pneumococci (Philips et al., 2003). Once the pneumococci escapes the nasopharynx and invades into the lung and blood, it encounters high glucose concentrations, thus the maximum CPS biosynthesis is needed (Baker et al., 2007). It implies that glucose concentration maybe impact the regulation of CPS production. However, it is unclear that the glucose concentration involves in regulating the expression of ComE. In this study, we have detected the CPS production and the expression of ComE in D39-WT and D39ΔcomE mutant grown in different glucose concentration medium. The results showed that the expression of ComE decreased significantly with the increasing glucose concentration from 2.0 to 16.0 mM (^*^p < 0.05; Figure 5A). Corresponding to the expression of ComE, the CPS production in D39-WT elevated significantly with the increasing glucose concentration from 2.0 to 8.0 mM. What's more, the CPS production in these concentration groups of D39ΔcomE mutant was significant higher compared to that of D39-WT (^***^p < 0.001, Figure 5B). These results demonstrated that extracellular glucose concentrations could impact the expression of ComE and regulate positively the CPS production. However, we also noted that the CPS production of D39ΔcomE mutant only slight increased with increasing glucose concentration (Figure 5C)., suggesting there should be other regulatory factors involved in the regulation of glucose on CPS production except of ComE.

### Deletion of ComE in D39 reduces nasopharyngeal colonization and leads to virulence enhancement in mice pneumonia and bacteremia models

**Figure 6:**
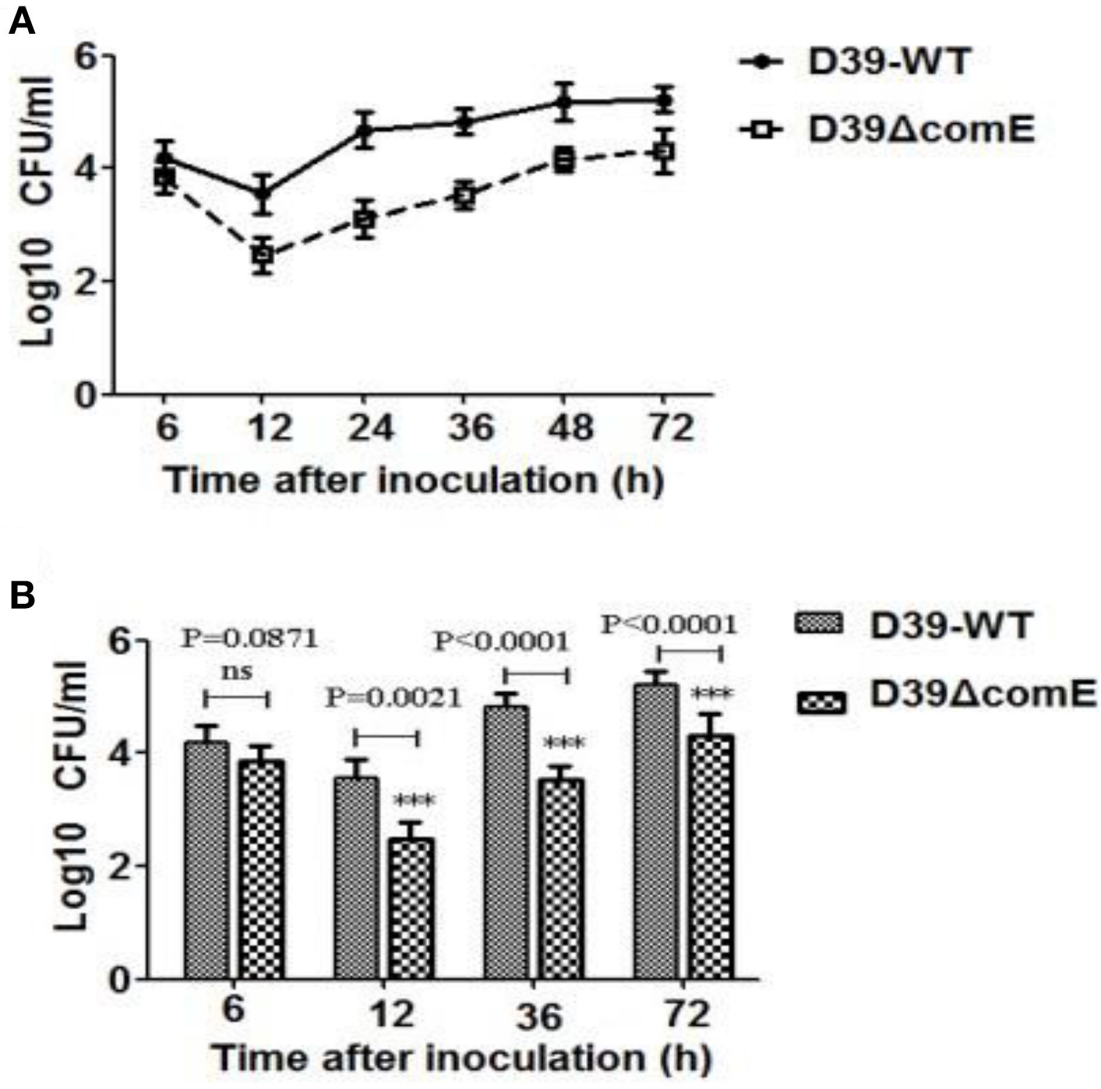
**The role of ComE in nasopharyngeal colonization. (A)** Nasopharyngeal colonization of Babl/c mice infected with the D39-WT and the D39ΔcomE mutant. Mice (*n* = 18) were intranasally challenged with 30 μl pneumococci (3.0 × 10^6^ CFU). Colonized bacteria were harvested from nasal lavage fluid and enumerated by plating serial dilutions. The graphs show log10 CFUs (SD) recovered from nasopharynx of three Balb/c mice for D39-WT or D39ΔcomE mutant. **(B)** Comparison of the number of colonized bacteria between in D39-WT and D39ΔcomE mutant group from the results represented in **(A)**. Statistical difference was determined by unpaired two-tailed Student's *t*-test. ^***^P < 0.001; ns, no significant difference.

As mentioned above, ComE impacts the capsule production by down-regulation of the *cps* locus transcription, and the capsule is the most important virulence factors for effective colonization and invasive infections. Therefore, we hypothesized that ComE could impact pneumococcal colonization and virulence. We found that the number of colonized bacteria tended to be stable after inoculation 48 h, and the number of colonized bacteria in D39-WT group was about 8–10 times that of in D39ΔcomE mutant group (Figure 6A). Compared with D39-WT group, D39ΔcomE mutant group showed significant decrease in number of colonized bacteria after inoculation: 12, 36, and 72 h (Figure 6B). These results suggest that ComE-regulated capsular play an important role in pneumococcal colonization of the nasopharynx in mice.

**Figure 7:**
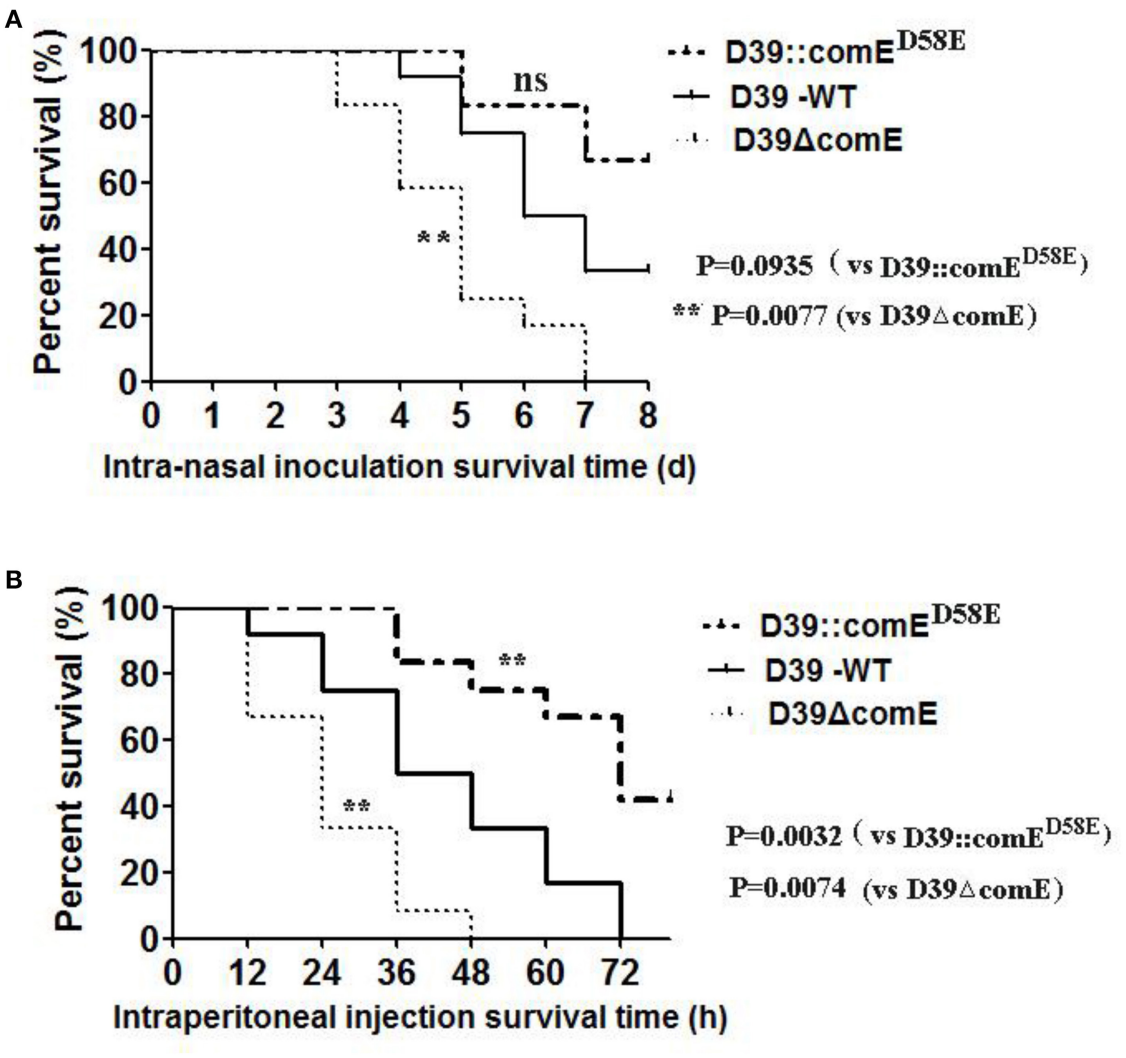
**The role of ComE in the virulence of ***S. pneumonia***e. (A)** Survival of mice during pneumococcal lung infection. Groups of Balb/c mice (*n* = 12) were challenged intranasally with D39-WT, D39ΔcomE and D39::comE^*D*58*E*^ mutant using 7.5 × 10^7^ CFU of bacteria. Mice were subsequently monitored for 8 days, and survival was recorded every day. D3-WT vs. D39ΔcomE, ^**^p = 0.0077; D39-WT vs. D39::comE^D58E^, no significant difference, *p* = 0.0935). **(B)** Survival of mice during pneumococcal bacteremia. Groups of Balb/c mice (*n* = 12) were administered with D39-WT, D39ΔcomE and D39::comE^D58E^ mutant by intraperitoneal injection using 7.5 × 10^2^ CFU of bacteria. Mice were subsequently monitored for 78 h, and survival was recorded every 12 h. D39-WT vs. D39ΔcomE, ^**^p = 0.0074; D39 WT vs. D39::comE^D58E^, ^**^p = 0.0032).

We also investigated the virulence of D39ΔcomE mutant in mice pneumonia and bacteremia models. In the pneumonia infection model, none of the D39ΔcomE mutant- infected mice (0/12) survived for the duration of the experiment, but 33.3% of the D39-WT –infected mice (4/12) survived until the end of the experiment. As shown in Figure 7A, the D39ΔcomE mutant was more virulent compared with the D39-WT strains (Log-rank test, *P* = 0.0077), but the over expression of ComE strains (D39::comE^D58E^) showed the equivalent virulence to that of D39-WT strains (Log-rank test, *p* = 0.0935) in the mice pneumonia model. In the bacteremia models, however, the D39::comE^D58E^ strains showed the weakest virulence. (Log-rank test, D39-WT vs. D39ΔcomE, *p* = 0.0074; D39-WT vs. D39::comE^D58E^, *p* = 0.0032; Figure 7B). Taken together, these results demonstrate that ComE affects the pathogenicity of D39 during infection *in vivo*.

## Discussion

Capsular polysaccharide is an important virulence factors required for effective pneumococcal infection (Kadioglu et al., 2008; Yother, 2011). The regulation of capsular production is essential for *S. pneumonia*e to survive in different niches of their hosts. Maximal expression of capsule is necessary for systemic virulence, but decreased capsule is beneficial for the adherence and colonization in the nasopharyngeal tract of the host (Hammerschmidt et al., 2005). Transcriptional regulation of the *cps* locus is recognized to be an efficient way to modulate pneumococcal CPS production (Shainheit et al., 2014; Wen et al., 2015; Wu et al., 2016). Previous work by Shainheit *et al*. has highlighted the role of transcriptional regulation as a potential mechanism of controlling capsule expression and uncovered the importance of the core promoter of capsular operon of *S. pneumonia*e in transcriptional regulation of capsular production (Shainheit et al., 2014). Another study by Wen et al. has revealed the necessary of the sequence elements upstream of the core promoter for the full transcription of the capsule gene operon (Wen et al., 2015). Our previous work has also screened several possible transcriptional regulators of *cps* locus (Figure S2), and confirmed that CpsR, a GntR family regulator, is a transcriptional regulator of *cps* locus (Wu et al., 2016). In this study, we further revealed that ComE, as a transcriptional regulator, involved in the transcriptional regulation of *cps* locus and capsular production.

To examine the role of ComE as an essential transcriptional regulator controlling capsule expression, we constructed D39ΔcomE, D39ΔcomE::comE^D58E^, and D39::comE^D58E^ mutants. As expected, Our results indicated that the deletion of ComE could increase significantly the transcription of the *cps* locus and CPS production, but when the deleted *comE* gene was ectopic complemented with ComE^D58E^ the transcription of the *cps* locus and CPS production restored the similar level to that of D39-WT strains. It was puzzling that the CPS production of D39::comE^D58E^ was not further reduced drastically with the increasing overexpression of ComE^D58E^ (fine control of GFP+-ComE^D58E^ expression using the Zn2^+^-inducible *P*_CZCD_ promotor, in Figure 3A). This could be partly explained that ComE reached a certain concentration that was out of the saturation of ComE binding site to *cps* locus the regulating effect of ComE on CPS production was gradually decreased with the increasing ComE concentration.

It is well documented that the phosphorylation of ComE plays an important role during the induction of competence for gentic transformation of *S. pneumoniae* and that ComE/ComE~P interplay dictates activation or extinction status of pneumococcal competence (Ween et al., 1999; Martin et al., 2013). In this study, we also demonstrated that only ComE~P has the activity of regulating CPS production by EMSA of ComE^D58E^ binding to *cps* promoter *in vitro* (Figure 1C) and by CPS analyses of D39ΔcomD mutant *in vivo* (Figure S4). We constructed a phosphoryl mimetic mutant ComE^D58E^ to realize the function simulation of ComE~P because of the failure to detect the transphosphorylation of purified ComE *in vitro* (Figure S3). We also constructed the D39ΔcomD and D39ΔcomD::comE^*D*58*E*^ mutants to demonstrate the impact of phosphorylation of ComE on the CPS production. The deletion of comD gene led to a non-phopshorylatable version of ComE in D39ΔcomD strain, which increased significantly the CPS production compared with that of D39-WT, as the D39ΔcomE strain did. When the D39ΔcomD strain was ectopic complemented with ComE^D58E^ the CPS production restored the similar level to that of D39-WT strains. Our results indicated that the phosphorylation of ComE did impact on the regulation of CPS production.

ComE has been characterized in detail as the master competence switch in transformation of *S. pneumoniae* (Pestova et al., 1996; Guiral et al., 2006; Martin et al., 2013; Weng et al., 2013). Several recent studies have shown that CSP-ComD/E competence system is not only essential for competence, but also important for the regulation of virulence factors required for infection (Ibrahim et al., 2004; Kowalko and Sebert, 2008). The CSP-ComD/E competence system influences the pneumococcal colonization of respiratory tract, and competence-mediated cell lysis may mediate the release of the virulence factors lipoteichoic acid (LTA) and pneumolysin (Guiral et al., 2005; Claverys et al., 2007). In the present study, we confirmed that the deletion of ComE in D39 reduces nasopharyngeal colonization and leads to virulence enhancement in mice pneumonia and bacteremia models. Previous study by Kowalko and Sebert has shown that deletion of the ComE increases fitness for colonization in an infant rat competitive colonization model of asymptomatic carriage (Kowalko and Sebert, 2008). However, we observed an inconsistent result that deletion of the ComE reduces fitness for colonization instead of increasing in an adult Balb/c mice colonization model. The increased expression of CPS in D39ΔcomE mutant will hinder cytoadherence, which may account for their inefficiency at nasopharyngeal colonization. Thicker capsular limits the extent of exposure of important pneumococcal surface structures and decreases the adherence ability to respiratory epithelial cells, which influences the following colonization in nasopharyngeal of mice (Talbot et al., 1996; Hammerschmidt et al., 2005; Voss et al., 2012). In addition, the inconsistent colonization result could be explained in part for the different immune status between newborn Sprague- Dawley rat and adult Babl/c mice.

The deletion of ComE also leaded to virulence enhancement in mice pneumonia and bacteremia models, which was attributed to that thicker capsular of D39ΔcomE mutant increased resistance to opsonophagocytosis (Figure 7). In the bacteremia models, the D39::comE^D58E^ strains showed weaker virulence, which was consistent with its' the thinner capsular than D39ΔcomE mutant. Because ComE regulates negatively CPS production and leads to declined virulence of *S. pneumoniae*, it is possible that ComE or ComE analogs may be applied to attenuate virulence of *S. pneumonia*e induced infections.

It has long been recognized that CPS reduces the natural competence of the pneumococcus for genetic transformation (Ravin, 1959; Weiser and Kapoor, 1999). The encapsulation of the pneumococcus acts as a barrier that prevents the competence-stimulating (CSP) from reaching its cellular target (Yother et al., 1986). Previous work by Schaffner et al. had shown that the expression of competence pathway genes was 11–34-fold higher and transformation frequency was 3.7-fold greater in non-encapsulated variant than the encapsulated (Schaffner et al., 2014). These studies have demonstrated that CPS could impact the development of competence in the pneumococcus genetic transformation. Therefore, we hypothesized that the regulation of ComE-mediated CPS production was associated with the development of competence. We used CSP1 as an exogenous inducer to examine the role of CSP-ComD/E competence system on the capsule production. Interesting, we found that CSP-ComD/E competence system involved in regulating negatively the CPS production in the development of competence D39 (Figure 4). It has been reported that pneumococcal surface structures, such as the type IV pilus (T4P) system, is essential for DNA binding and import in *S. pneumoniae* (Laurenceau et al., 2013). Given the main objective of bacterial transformation for the uptake of exogenous genes, the thinner capsular facilitates exposure of the type IV pilus (T4P) system and enhances the exogenous DNA to import into the periplasm from the extracellular side during transformation of *S. pneumonia*e. Obviously, it is benefit for the progress of bacterial transformation that the CSP-ComD/E competence system regulates negatively the ComE-mediated CPS production.

A carbon source might affect CPS expression. Previous work by Giammarinaro and Paton has shown that the RegM, involving in the regulation of sugar-metabolism pathways, might impact transcription of the *cps* locus (Giammarinaro and Paton, 2002). The other two proteins involving in sugar metabolism, Pgm catalyzing the conversion of glucose-6-phosphate to glucose-1-phosphate and GalU catalyzing the formation of uridine diphosphate-glucose from glucose-1-phosphate, have been shown to affect CPS production (Mollerach et al., 1998; Hardy et al., 2001). Mutants of *S. pneumoniae* in which either the *galu* or *pgm* gene was disrupted produced almost no CPS and exhibited growth defect (Mollerach et al., 1998; Cieslewicz et al., 2001). Glucose is an important nutrient factor for pneumococcal growth and metabolism (Philips et al., 2003; Brennan et al., 2007). Very low level of glucose is detectable in healthy pharynx nasalis, where minimal expression of CPS is required for optimal adhesion and colonization of pneumococci (Philips et al., 2003). Once the *S. pneumoniae* escapes the nasopharynx it encounters high glucose concentrations into the lungs and blood, thus the maximum expression of CPS is essential for systemic virulence (Baker et al., 2007). The glucose concentrations maybe, as a specific environmental signal, play a role in regulating CPS production. Our results indicated that ComE involve in regulating the expression of CPS responding to extracellular glucose signal (Figure 5). We would need to make a further study over time to determine this mechanism.

The DNA affinity chromatography-pulldown is well established effective methods to isolate and identify novel transcriptional regulator, which do not demand any knowledge of the DNA-binding protein's identification (Jutras et al., 2012). In this study, several possible proteins have been isolated and identified as candidate transcriptional regulators using *cps* promoter DNA as bait: ComE, CcpA, hypothetical protein SPD_0410, DNA-binding protein HU, GntR, PlcR and MarR (Figure S2). Of note, previous work by Giammarinaro and Paton has revealed that CcpA appears to be involved in transcriptional activation of the *cps* operon in the D39 strain (Giammarinaro and Paton, 2002), but it was still not confirmed as the transcriptional regulator of *cps* locus. So far, we have confirmed that GntR (CPSR) and ComE are transcriptional regulators of *cps* locus. Despite it has been demonstrated that both GntR and ComE regulate negatively the CPS production, it is worthy of further study whether there is some correlations in their regulation function.

## Conclusion

Our results show that *S. pneumonia*e D39 ComE negatively regulates the transcription of *cps* locus and impacts the CPS production, which leads to the attenuation of the virulence in pneumococcal. CSP-ComD/E competence system involves in capsular regulation in the progress of natural genetic transformation of *S. pneumonia*e. Extracellular glucose may affect the expression of ComE and regulate positively CPS production. Given the importance of capsule in pneumococcal virulence and invasive infection, and the regulation of CSP-ComD/E system on CPS production, it deserves to be further studied that CSP may be applicable to reduce the invasive infections by *S. pneumoniae*.

## Author contributions

Conceived and designed the experiments: YZ, XZ, and YY. Performed the experiments: YZ, XW, LW, and JZ. Analyzed the data: YZ and XZ. Contributed reagents/materials/analysis tools: YZ, XZ, and XW. Wrote the paper: YZ and YY. All authors read and approved the final manuscript.

### Conflict of interest statement

The authors declare that the research was conducted in the absence of any commercial or financial relationships that could be construed as a potential conflict of interest.

## Abbreviations

CPS: capsular polysaccharide
EMSA: electrophoretic mobility shift assay
C+Y: semisynthetic casein hydrolysate medium supplemented with 5% yeast extract
LB agar: Luria-Bertani medium
GFP: green fluorescent protein
SDS-PAGE: SDS- polyacrylamide gel electrophoresis
CSP: competence-stimulating peptide
GAPDH: Glyceraldehyde-3-phosphate dehydrogenase.
