# Supplementary material for: ComE, an Essential Response Regulator, Negatively Regulates the Expression of the Capsular Polysaccharide Locus and Attenuates the Bacterial Virulence in Streptococcus pneumoniae
Source: Front Microbiol. 2017 Mar 7;8:277. doi: 10.3389/fmicb.2017.00277 (PMC5339220; doi:10.3389/fmicb.2017.00277)
Supplement: Supplementary file 1 [file Image1.pdf]

Figure S1 Genetic architecture of the *cps* locus in *S. pneumoniae*. **(A)**The locus shown is representative of the Wzy-dependent capsule cassette of serotype 2. The capsule locus, ~15 kb in size, is located between the conserved genes *dexB* and *aliA* and is flanked on either side by insertion sequences (IS). *Cps2A-2D* are highly conserved across most serotypes. Serotype-specific genes encode all enzymes required to synthesize serotypespecific capsular polysaccharides. **(B)** Sequences of the promoter for D39. The transcriptional start site for the promoter P*cps* is assigned +1 and other positions are numbered accordingly. The initiation codon of cps2A is indicated with an arrow. The primer pair of P_{cps}-F and P_{cps}-R is indicated with an arrow underline, which is used to prepare C2 probe for DNA affinity chromatography-pull down analysis.

Figure S2 Isolating and identifying binding proteins to *cps* promoter. **(A)** SDS-PAGE analysis of *S. pneumoniae* cytoplasmic proteins fished out by affinity chromatography using *cps* promoter DNA probe (C2) as bait. M, mass maker; Lane c1 and c2 were eluted with NaCl at 100mM and 200mM under inducing conditions, and lane k1 and k2, as blank magnetic beads control, respectively. Proteins were stained with Coomassie Blue Staining. Numbers on the lane indicate the enriched proteins excised and identified by MAIDI-TOF mass spectrometry. **(B)** The partial MS result proteins that are most likely chosen to be further studied. **(C)** The distribution of coverage rate of 24 proteins from the MS results. **(D)** Verifying the specific binding ability of candidate proteins to *cps* promoter by EMSA *in vitro*. The results showed that GntR,Tr-act,MarR,HU,0410,andCcpA can bind specifically to *cps* promoter, but non-phosphorylated ComE can bind hardly to it.

Figure S3 Mimetic phosphorylated ComE. **(A)** Shows the amino acid sequence of ComE. The enriched protein from the 13 sample is identified as response regulator ComE with 78.4% coverage rate by MALDI-TOF MS analysis. The consistent amino acids sequence with MS is indicated with red italic underline text. **(B)**Phosphorylated mimetic mutant ComE^{D58E} is constructed by site-directed mutagenesis of comE *in*

vitro. The molecular structure of mimetic site amino (Glu) is showed above the nucleotide of comE. The site-directed mutagenesis nucleotide (G) and primer sequence are indicated with box and red underline, respectively. **(C)**EMSA of ComE^{D58E} binding to *cps* promoter sequence (C2 probe).The result shows that the ComE^{D58E} exhibits significantly improved binding affinity for *cps* promoter sequence.

Figure S4 The role of phosphorylation of ComE in regulating the CPS production. (A) Identification of D39ΔcomD、D39ΔcomE、D39ΔcomD**::**comE$^{D58\mathbf{E}}$ and D39ΔcomE**::**comE$^{D58\mathbf{E}}$ mutants by PCR. **(B)** Detection of CPS production in D39 and its mutants by Western blot. GAPDH is detected as an internal control for constitutively expressed gene. The ratio of gray values of each sample CPS to GAPDH is calculated. The results of representative experiments are presented as means of three replicates ± standard deviations. **, $P<0.01$. （**C**） Neufeld test for D39-WT and its mutants. The mean capsule thickness from 100 swelling cells is measured with Image J software:D39-WT (0.4678±0.0985μm) 、D39ΔcomE (0.6271±0.1343μm) 、 D39ΔcomD (0.6530±0.1149μm) and D39ΔcomD::comE^{D58E}(0.4778±0.0846μm).The statistical result shows that the average swelling capsule thickness of D39ΔcomE mutant and D39ΔcomD are significantly increased compared with that of D39-WT (**, $p<0.01$). These results indicate that the phosphorylation of ComE impact on the CPS production.

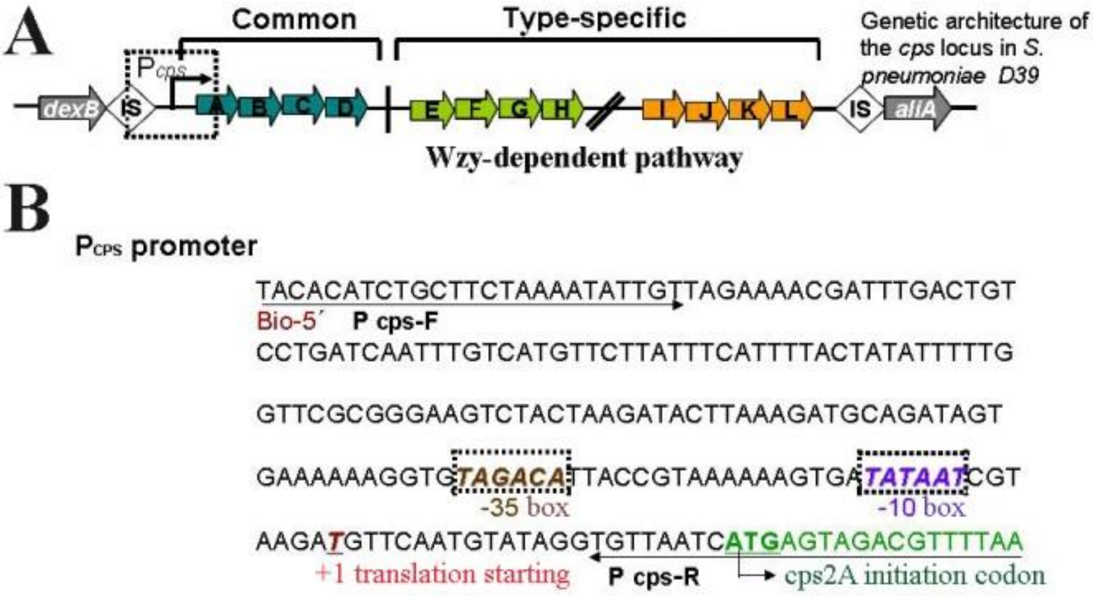

Figure S1 JPEG

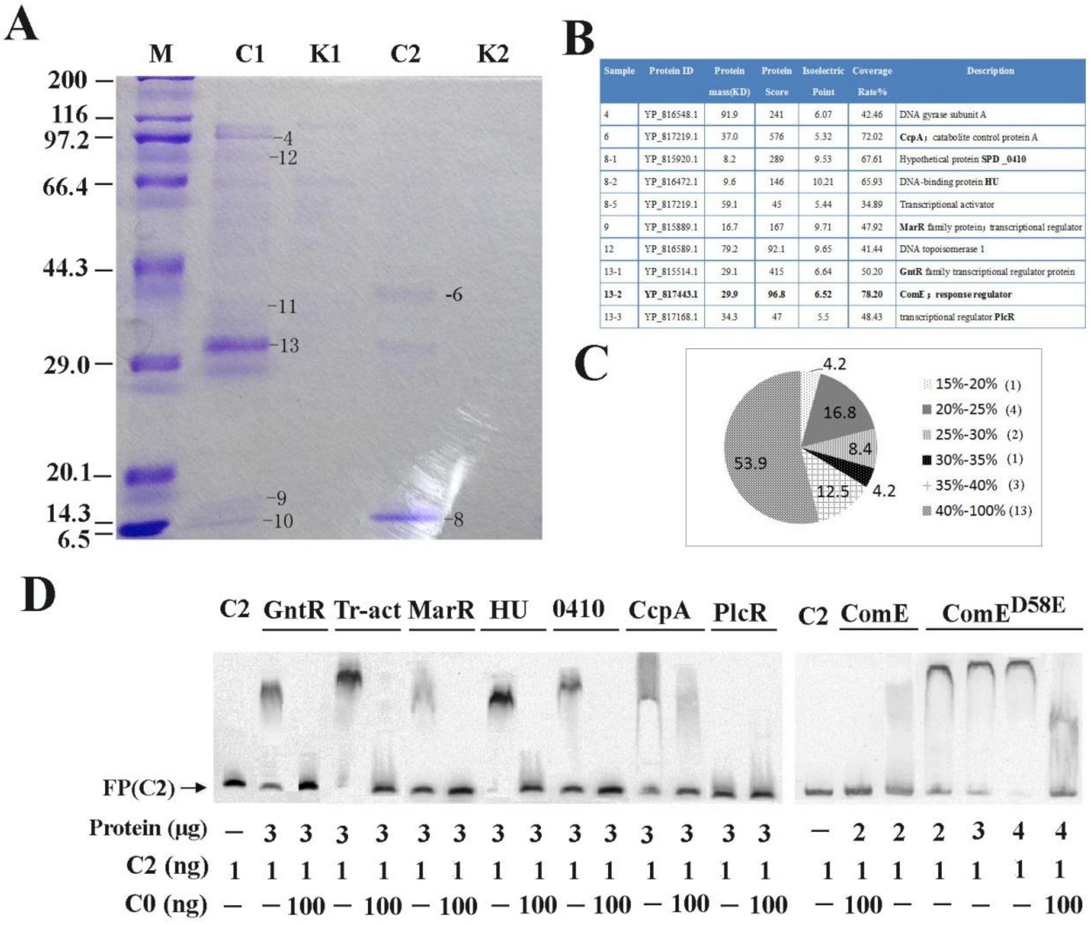

Sample	Protein ID	Protein mass(KD)	Protein Score	Isoelectric Point	Coverage Rate%	Description
4	YP_816548.1	91.9	241	6.07	42.46	DNA gyrase subunit A
6	YP_817219.1	37.0	576	5.32	72.02	**CcpA**； catabolite control protein A
8-1	YP_815920.1	8.2	289	9.53	67.61	Hypothetical protein **SPD _0410**
8-2	YP_816472.1	9.6	146	10.21	65.93	DNA-binding protein **HU**
8-5	YP_817219.1	59.1	45	5.44	34.89	Transcriptional activator
9	YP_815889.1	16.7	167	9.71	47.92	**MarR** family protein; transcriptional regulator
12	YP_816589.1	79.2	92.1	9.65	41.44	DNA topoisomerase 1
13-1	YP_815514.1	29.1	415	6.64	50.20	**GntR** family transcriptional regulator protein
**13-2**	**YP_817443.1**	**29.9**	**96.8**	**6.52**	**78.20**	**ComE ; response regulator**
13-3	YP_817168.1	34.3	47	5.5	48.43	transcriptional regulator **PlcR**

Figure S2. JPEG

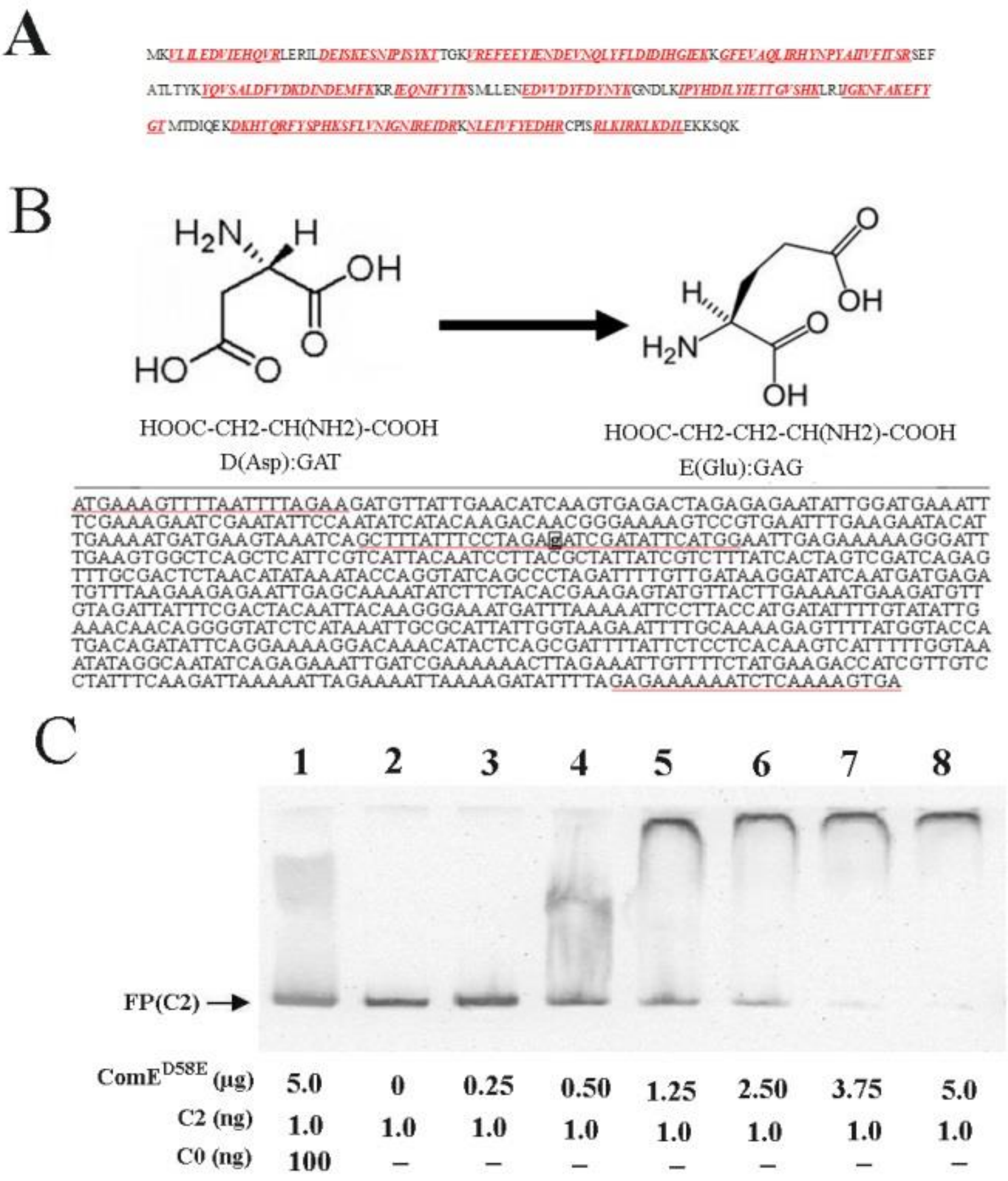

Figure S3. JPEG

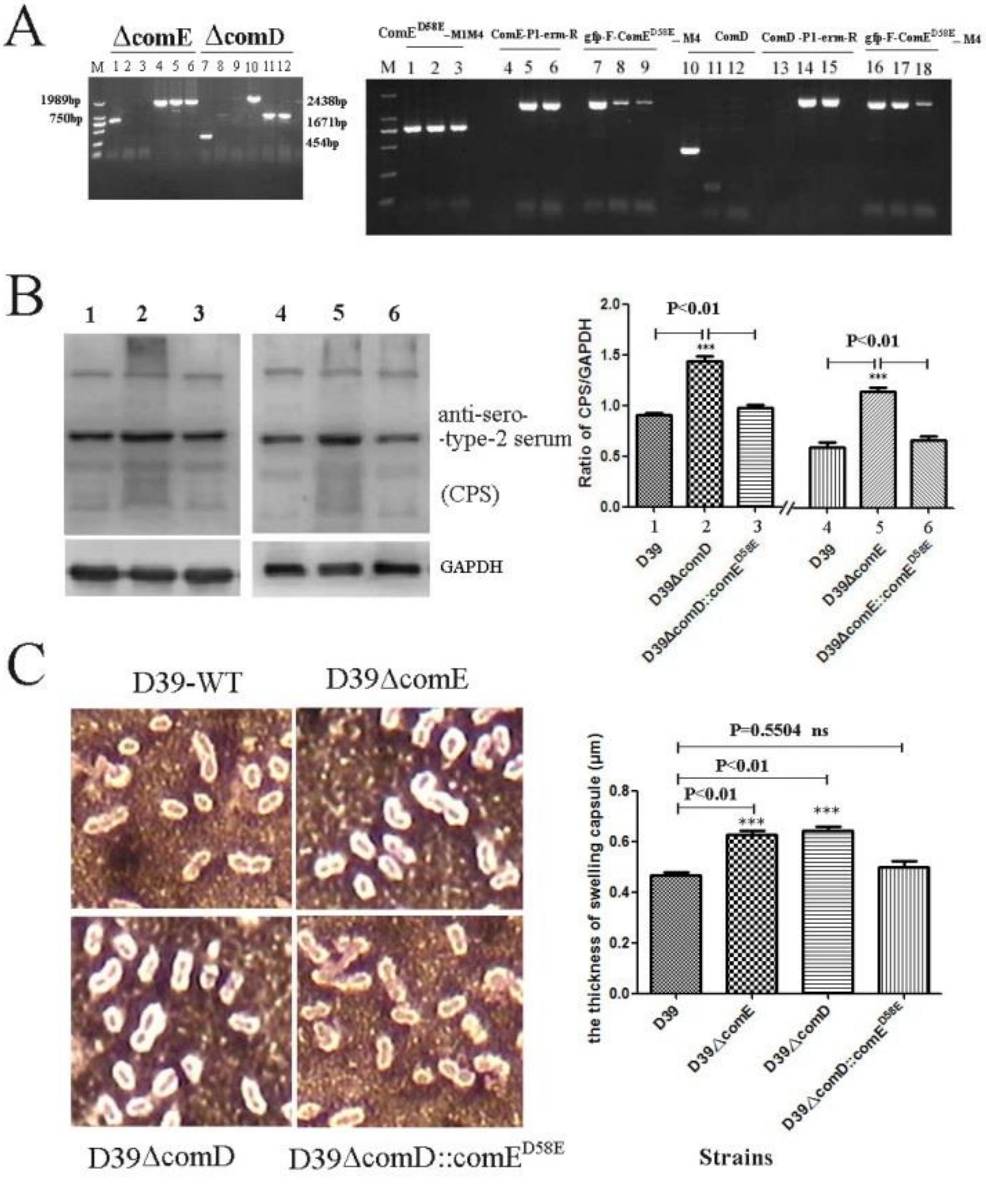

Figure S4. JPEG
